# Social and behavioral vulnerability, pregnancy, and negative mental health outcomes in the U.S. during the Covid-19 pandemic

**DOI:** 10.3934/publichealth.2022023

**Published:** 2022-02-21

**Authors:** Stephanie A. Godleski, Casey T. Harris, Kevin M. Fitzpatrick, Ammina Kothari

**Affiliations:** 1 Department of Psychology, Rochester Institute of Technology, 92 Lomb Memorial Drive, Rochester, NY 14623, USA; 2 Department of Sociology and Criminology, University of Arkansas, 211 Old Main, Fayetteville, AR 72701, USA; 3 Harrington School of Communication and Media, University of Rhode Island, Davis Hall, Kingston, RI 02881, USA

**Keywords:** Covid-19, negative mental health, pregnancy, worry, social vulnerability, risks

## Abstract

The novel coronavirus (Covid-19) pandemic has had a significant impact on the mental health of the general U.S. population. Extant literature has increasingly linked social vulnerabilities, risky behavior, and limited social and psychological resources to the growing mental health crisis during the virus's spread. The purpose of this study was to examine the impact of pertinent social vulnerabilities and subjective risk factors for both men and women on mental health (i.e., depression, anxiety, isolation) with a closer examination of the influence of pregnancy during the pandemic on mental health. The sample included 740,640 respondents participating in the U.S. Covid-19 Trends and Impact Survey that was deployed between February and March 2021. Descriptive statistics and ordinary least squares regression models are presented with a focus on the factors that shape negative mental health outcomes, particularly on the disparities between pregnant and non-pregnant women relative to men, but also subjective/perception factors (e.g., fear/worry) and social vulnerabilities. Results show that pregnant women were at significantly greater risk of negative mental health outcomes at this stage of the pandemic than either men or non-pregnant women. Overall, respondents who were younger, without children in the household, unemployed, worried generally about infection or their finances, or had ever tested positive for Covid were also more likely to report feelings of anxiety, isolation, and depression than their counterparts. Pregnant women may be especially vulnerable to depression, anxiety, and isolation during the pandemic and our findings suggest the importance of developing targeted mental health support for this sub-population.

## 1. Introduction

Since the early days of the Covid-19 global pandemic, a growing body of empirical research has documented the profound impact of the pandemic on the psyche of citizens around the world, with several negative mental health outcomes having been identified as consequences of the Covid-19 pandemic for the general U.S. population (e.g., [1]–[3]). This has been true even among those who have no previous history of mental health problems [4]. For example, evidence from a poll in the early months of the pandemic found that nearly half of U.S. adults surveyed reported their mental health was being negatively impacted because of worry, stress, and anxiety caused by the coronavirus [5]. Indeed, the severe distress brought on by the pandemic has already been found to be associated with suicidal thoughts and behaviors [6],[7]. Other scholarship reveals the varied social, cultural, economic, and psychological factors that have had a significant impact on the mental health sequelae of the general population (e.g., [3],[8],[9]). Still other scholars find that pandemic prevention and/or mitigation strategies (e.g., social distancing, remote work/learning) may have further exacerbated mental health issues by fostering isolation, loneliness, and stress [10],[11]. With the emergence of new variants, understanding which specific risk factors are tied to negative mental health outcomes during the Covid-19 pandemic remains paramount.

We focus, in particular, on the growing literature demonstrating how social vulnerability captured by a host of sociodemographic characteristics are related to higher distress and negative health outcomes [12],[13]. This emerging body of empirical work reveals that, while men may be more susceptible to severe illness from Covid-19 [14], women appear to be at higher risk for experiencing and reporting poorer mental health status [9],[13]. Similarly, those who are younger, have lower levels of education [8],[13], who worry about family finances [4], those with children present, and individuals that worry about infection from Covid-19 itself [2],[15] report more negative mental health outcomes.

Considerably less attention has been devoted to the complexity of Covid-19 related mental health among families, including those expecting children during the pandemic. Our focus centers on whether pregnant women are at greater risk for poor mental health outcomes because parents' mental well-being has been particularly impacted by a wide-range of stressors during the pandemic, including disruptions to work-life balance and additional caregiving responsibilities (see [16]). This may be especially true for mothers, who have already emerged as an at-risk group [17],[18]. For example, Moyer and colleagues found that rising tension in the home, worry about getting infected while pregnant, and other factors were associated with pregnancy-related anxiety [19]. Nevertheless, how pregnancy during the pandemic impacts mental health more broadly, especially in comparison to other pertinent risk factors, remains under-examined. Thus, the aim of the present study is to examine the impact of social and demographic vulnerabilities, including pregnancy, as well as individual perceptions of risk from infection or household financial strain, and personal experience with Covid-19 and vaccination as they impact mental health (depression, anxiety, and isolation) within the general adult United States population.

The current study leverages data collected by the Delphi Group at Carnegie Mellon University for the U.S. Covid-19 Trends and Impact Survey, in partnership with Facebook (for additional information on this project, see [20]). This larger study examines the effects of Covid-19 throughout the United States using a voluntary, web-based survey advertised and distributed daily on Facebook. As described below, the data used in the present study are especially valuable because they capture mental health outcomes as vaccines were just beginning to become widespread in the United States and before we observed major divergence across political and social lines regarding vaccine intent [21]. Examining this period provides a clearer picture of the unique psychological concerns surrounding pregnancy and other social vulnerabilities less confounded by the subsequent debate about vaccine side effects and verification of vaccination status [22].

## 2. Materials and methods

The 8th Wave of the U.S. Covid-19 Trends and Impact Survey (CTIS) collected responses from Facebook users aged 18 and above (n = 1,277,961) through a web-based survey that rolled out beginning in February 8, 2021 to March 2, 2021. During this period on each day, a new randomly stratified sample of active Facebook users aged at least 18 years older in the United States were invited to participate in the study through a survey invitation displayed at the top of their News Feed [23]. This invitation linked to the Delphi US CTIS on Qualtrics. We restricted analysis to the 740,640 respondents for whom socio-demographic vulnerabilities, mental health, and Covid-19 related worry and testing measures were provided (i.e., non-missing). Participants provided consent and the survey protocol was reviewed by the Carnegie Mellon University Institutional Review Board (for additional details on this survey, see [23],[24]).

As the central focus of the analysis, pregnancy is cross classified with gender using measures for pregnant women and non-pregnant women whereby men serve as the reference category. Additionally, we include a set of social vulnerabilities as follows: age, currently employed, level of education, and whether the household had children present. In addition, participants reported how worried they were that they or someone in their “immediate family might become seriously ill from Covid-19” (Worry – Covid) and how worried about “household's finances for the next month” (Worry – Finances). Responses for the latter two ranged from 1 = “not at all worried” to 4 = “very worried”. Participants also reported whether they were they had ever tested positive for Covid-19 and whether they had received all doses of the Covid-19 vaccine at the time of survey. The primary dependent variable was measured by asking respondents how often in the last five days they felt “nervous, anxious, or on edge”, “depressed”, and “isolated from others”. Each response was captured using a scale ranging from 1 = “none of the time” to 4 = “all of the time”.

We estimate a series of linear regression models to examine the role of pregnancy and our selection of social and behavioral vulnerabilities as they are related to the mental health problems scale. Model 1 includes the focal pregnancy and gender measures, as well as key social vulnerabilities (demographic controls). Model 2 introduces additional measures of subjective worry and risk regarding Covid-19 and finances, while Model 3 is saturated with the inclusion of the final vaccine participation measure. All models are estimated with robust, zip code-clustered standard errors to account for shared variance among respondents from the same geographic locations. This reduces the likelihood that associations shared among respondents from the same communities (zip codes) biases statistical significance testing.

## 3. Results

**Table 1.** Summary statistics of model variables (n = 740,460).

	%	Mean	SD
*Mental Health Items ^a^:*	-	5.15	2.13
Anxious	-	1.73	0.84
Depressed	-	1.61	0.79
Isolated	-	1.81	0.88
*Gender by Pregnancy:*			
Non-Pregnant Woman	64.89	-	-
Pregnant Woman	0.14	-	-
Man	34.97	-	-
*Other Social/Demographic Vulnerabilities:*
Age (18–24) ^b^	4.42	-	-
Age (25–34) ^b^	12.74	-	-
Age (35–44) ^b^	16.03	-	-
Age (45–54) ^b^	17.01	-	-
Age (55–64) ^b^	20.46	-	-
Age (65–74) ^b^	20.68	-	-
Age (75+) ^b^	8.66	-	-
Employed	38.21	-	-
Education (<high school) ^b^	3.06	-	-
Education (high school graduate) ^b^	16.32	-	-
Education (some college) ^b^	24.76	-	-
Education (2 years degree) ^b^	11.58	-	-
Education (4 years degree) ^b^	24.36	-	-
Education (Master's degree) ^b^	14.33	-	-
Education (professional degree) ^b^	3.29	-	-
Education (doctorate) ^b^	2.31	-	-
Child Present	32.61	-	-
Worried – Covid	-	1.78	0.93
Worried – Finances	-	1.31	1.08
Ever Covid Positive	44.91	-	-
Received All Vaccine Doses	0.96	-	-

*Note: ^a^ Cronbach's alpha for the scale items is 0.81 with inter-item correlations ranging from 0.51 to 0.66. ^b^ Age and education are displayed categorically here for ease of interpretation but are treated as continuous covariates in subsequent models.

Most respondents in the sample identified as women and had at least some college education. Respondents were evenly distributed across age categories. About one-third of respondents had children present at the time of survey, and less than half reported having worked in the four weeks prior. Half of respondents tested positive for Covid-19 at some point during the pandemic, a pattern that mirrors the estimates provided by the Centers for Disease Control and Prevention [25]. The average respondent scored moderate with regards to mental health problems (mean = 5.15; SD = 2.13) with isolation being subjectively reported as somewhat more likely than either feeling depressed or anxious. The three questions on negative mental health were highly correlated (with scores ranging from 0.51 to 0.66) and summed to create a composite mental health problems scale (α = 0.81 with scores ranging from 3 to 12).

Model 1 in the regression table reveals that, relative to men, women – both pregnant and non-pregnant – report more depression, anxiety, and/or isolation. Additionally, respondents that are younger, unemployed, and without children report poorer mental health than their counterparts, all other characteristics being held constant (p < 0.05). Critically, pregnant women emerge as doing especially poorly (b = 0.70, p < 0.001) even compared to non-pregnant women (b = 0.46, p < 0.001). Tests for the equality of coefficients (not shown) indicate this difference in coefficients is statistically significant.

**Table 2.** Zip-code clustered ordinary least squares regression of gender, pregnancy, and other social vulnerabilities on mental health scale (n = 740,460).

	Model 1	Model 2	Model 3
Pregnant Woman	0.70***	0.61***	0.61***
	(0.08)	(0.08)	(0.08)
Non-Pregnant Woman	0.46***	0.33***	0.33***
	(0.01)	(0.01)	(0.01)
Age	−0.39***	−0.32***	−0.32***
	(0.01)	(0.01)	(0.01)
Employed	−0.46***	−0.41***	−0.41***
	(0.01)	(0.01)	(0.01)
Education	0.01	0.08***	0.08***
	(0.01)	(0.01)	(0.01)
Child Present	−0.26***	−0.29***	−0.29***
	(0.01)	(0.01)	(0.01)
Worried – Covid	-	0.39***	0.39***
	-	(0.01)	(0.01)
Worried – Finances	-	0.41***	0.41***
	-	(0.02)	(0.02)
Ever Covid Positive	-	0.08***	0.08***
	-	(0.01)	(0.01)
All Vaccine Doses	-	-	−0.16***
	-	-	(0.02)
R^2^	0.09	0.17	0.17

*Note: Robust standard errors clustered by zip code shown in parentheses. * p < 0.05, ** p < 0.01, *** p < 0.001

Adding in other risk factors, Model 2 finds similar patterns for the key social vulnerability characteristics observed in Model 1, but also indicates that worry about infection to oneself or family, worry about finances, and having ever tested positive for Covid-19 are associated with more mental health problems (p < 0.001). At the same time, the inclusion of these additional subjective risk/worry measures attenuated the gender-by-pregnancy coefficients, but more substantially among non-pregnant women (reduced by approximately 28 percent) than for pregnant women (reduced by approximately 12 percent). Thus, pregnant women's mental health problems seem less attributable to worry about infection, family finances, and prior history of Covid-19 compared to women who are not pregnant.

Model 3 shows that receiving all vaccine doses reduces mental health problems. However, the association is comparably weak compared to other factors in the model. Admittedly, few respondents had received all doses at the time of survey in February-March of 2021. Nevertheless, mental health consequences seem to be driven more by subjective factors (e.g., fear/worry) and social vulnerability. While vaccine compliance is important, it does not attenuate the significant associations between social and subjective factors on negative mental health outcomes among the respondents in our sample, including among pregnant women.

## 4. Discussion

The present study examined the impact of social and demographic vulnerabilities, individual perceptions of risk from infection or financial strain, personal Covid-19 experiences, and vaccination compliance within the general adult United States population on subjective mental health assessments (depression, anxiety, and isolation). A particular focus of the study was to examine the role of gender by pregnancy status. Our findings revealed that women, especially pregnant women, reported more depression, anxiety, and/or isolation. In addition, participants who were younger, unemployed, and without children also had higher reported mental health concerns. Subjective factors (e.g., worry about infection to oneself or family, worry about finances) were also associated with increased reported mental health problems; while in contrast, having received all doses of the Covid-19 vaccine reduced mental health concerns.

Overall, results highlighted the importance of subjective factors, such as fear and worry, and social vulnerability. Consistent with past research [9],[13], women typically report higher levels of mental health problems (i.e., depression, anxiety, isolation) during the Covid-19 pandemic. This level of uncertainty and depression may be in part accounted for by other pertinent factors, such as concerns about infection within the family, financial worries, and personal experience with Covid-19. Importantly, as hypothesized, pregnant women reported higher levels of negative mental health concerns compared to both non-pregnant women and men. However, for pregnant women, this association was not as significantly impacted by other demographic or subjective factors. This unique effect of pregnancy may be due to a specific type of anxiety rooted in pregnancy-related exposure concerns during a pandemic (e.g., attending in-person prenatal care appointments; [19]), or even heightened uncertainty about the physical consequences of severe Covid-19 illness during pregnancy [26]. Further, prevention and mitigation practices, such as social distancing, may be especially impactful for pregnant women as social support is an important contributor to maternal health [18],[27]. The present study adds to the current literature by highlighting the unique mental health risk for pregnant women in the United States (e.g., [18]) and the need for additional social and psychological support during this particularly difficult period [28].

The relationship between full vaccination and reduced mental health problems is promising. However, rates of vaccination among pregnant women have been low in comparison to non-pregnant adult women of reproductive age [29] despite higher rates for the general population of women in comparison to men [30],[31]. Rates also appear to vary by age as well as race and ethnicity among pregnant women [29]. Therefore, reducing barriers and hesitancy to vaccination in support of increased vaccination coverage for pregnant women may be an additional strategy for potentially reducing anxiety and stress among this population.

Interestingly, our results showed that having children in the household did not increase mental health concerns, and instead those persons reporting no children in the household actually had higher rates of reported negative mental health outcomes. It may be that being a parent especially confers risk when in combination with other stressors or perceived challenges [16]. Alternatively, the initial acute strain of pandemic conditions and restrictions for families, such as pivoting to social distancing and homeschooling [32], may have exacerbated mental health problems, while having children as the pandemic continues may serve as a protective factor against loneliness, isolation, and depression [33],[34]. Future research should explore factors that may explain the association between having children in the home, or not, and mental health, including some delineation and assessment between those with intimate partner relationships and those without.

While these results are promising and provide additional support with a large national sample, it is not without limitations. Data for the current study reflect a critical and valuable period roughly one year after the declaration of a global pandemic, but prior to widespread vaccination. Nevertheless, more research is needed that examines the longitudinal patterns of mental health concerns impacting pregnant women and other vulnerable social groups into more recent weeks and months. Doing so would aid those looking to better understand the multidimensional circumstances and conditions responsible for exacerbating mental health problems during public health crises such that care providers might better allocate resources to mitigate the effects of those underlying circumstances. Future research should also incorporate clinical diagnoses of mental health concerns, as self-report may be biased (e.g., [36]), as well as more comprehensive, multifaceted assessments of severe psychological distress and suicidality [6],[7],[35]. Further, the proportion of pregnant women represented in the sample is relatively small, which future work should specifically target in recruitment and retention to further explore samples of pregnant women in terms of both statistical power and to expand inquiry into the complicated relationships underlying pregnancy and mental health during a public health crisis. In addition, whether women had changed or delayed their plans to become pregnant because of the pandemic was not assessed in the present study, though there is evidence that many women in the United States have reported delaying their plans to get pregnant or wanting to have fewer children due to the pandemic [37]. Indeed, birth rates decreased in 2020, particularly in the second half of the year [38],[39]. Finally, we note that the Delphi US CTIS does not provide information on individual responses regarding race and ethnicity to protect participants from potential identification; however, the mental health consequences of the pandemic may be intensified for underrepresented groups as observed in some studies to-date [40],[41]. Future research would do well to include efforts to further untangle the unique role of pregnancy on Covid-related mental health as it might differ across racial and ethnic groups, particularly as such research would better direct resources to those pregnant women most at risk. Importantly, understanding how social determinants of health may moderate these associations will provide additional information on factors that may further exacerbate mental health problems or impact access to resources, particularly as future research extends beyond the United States context alone and into international settings where pregnancy may carry different perceived and real risks (e.g., more/less prenatal and obstetric care, greater/lesser social support for pregnant mothers, different familial arrangements).

## 5. Conclusions

In conclusion, the results of the present study highlight the distinctive experience of depression, anxiety, and isolation for pregnant women during the pandemic in the United States, even in comparison to other substantively important factors impacting outcomes for both men and women. Results also suggest the importance of examining vulnerabilities within the context of other pertinent risk factors for mental health concerns, as worry about infection, family finances, and prior history of Covid-19 helped to explain the increased mental health concerns in women who were not pregnant, but did not as markedly account for concerns among expectant women. Intervention and prevention efforts targeting pregnant women may be especially salient in addressing the complicated mental health care for expectant parents. Further, given that having children in the home was associated with lower mental health concerns, first-time expecting women may be particularly vulnerable and benefit from additional intervention efforts. For pregnant women, enhancing social support [18],[27], addressing pregnancy-specific Covid-19 concerns [19],[26], and supporting increased vaccination coverage [29] may be helpful strategies for potentially reducing anxiety, stress, and isolation.
